# 5-Amino-1-naphthol

**DOI:** 10.1107/S1600536809045152

**Published:** 2009-10-31

**Authors:** Agnieszka Czapik, Arkadiusz Nitka, Maria Gdaniec

**Affiliations:** a Faculty of Chemistry, Adam Mickiewicz University, 60-780 Poznań, Poland

## Abstract

In the title compound, C_10_H_9_NO, the amino and the hydr­oxy groups act both as a single donor and a single acceptor in hydrogen bonding. In the crystal, mol­ecules are connected *via* chains of inter­molecular ⋯N—H⋯O—H⋯ inter­actions, forming a two-dimensional polymeric structure resembling the hydrogen-bonded mol­ecular assembly found in the crystal structure of naphthalene-1,5-diol. Within this layer, mol­ecules related by a translation along the *a* axis are arranged into slipped stacks *via* π–π stacking inter­actions [inter­planar distance = 3.450 (4) Å]. The amino N atom shows *sp* ^3^ hybridization and the two attached H atoms are located on the same side of the aromatic ring.

## Related literature

For the crystal structure of 1,5-dihydroxy­naphthalene, see: Belskii *et al.* (1990 ▶). For amino-hydr­oxy group recognition and packing motifs of aminols, see: Ermer & Eling (1994 ▶); Hanessian *et al.* (1994 ▶); Allen *et al.* (1997 ▶); Dey *et al.* (2005 ▶).
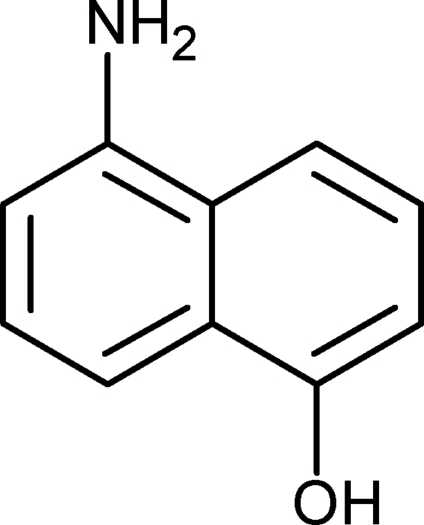


## Experimental

### 

#### Crystal data

C_10_H_9_NO
*M* *_r_* = 159.18
Orthorhombic, 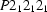

*a* = 4.8607 (2) Å
*b* = 12.3175 (6) Å
*c* = 13.0565 (5) Å
*V* = 781.71 (6) Å^3^
*Z* = 4
Mo *K*α radiation
μ = 0.09 mm^−1^
*T* = 130 K
0.30 × 0.15 × 0.02 mm

#### Data collection

Kuma KM-4-CCD κ-geometry diffractometer
Absorption correction: none
8484 measured reflections
963 independent reflections
726 reflections with *I* > 2σ(*I*)
*R*_int_ = 0.037

#### Refinement

*R*[*F* ^2^ > 2σ(*F* ^2^)] = 0.036
*wR*(*F* ^2^) = 0.091
*S* = 1.06
963 reflections
121 parameters
H atoms treated by a mixture of independent and constrained refinement
Δρ_max_ = 0.17 e Å^−3^
Δρ_min_ = −0.21 e Å^−3^

### 

Data collection: *CrysAlis CCD* (Oxford Diffraction, 2007 ▶); cell refinement: *CrysAlis RED* (Oxford Diffraction, 2007 ▶); data reduction: *CrysAlis RED*; program(s) used to solve structure: *SHELXS97* (Sheldrick, 2008 ▶); program(s) used to refine structure: *SHELXL97* (Sheldrick, 2008 ▶); molecular graphics: *ORTEP-3 for Windows* (Farrugia, 1997 ▶) and *Mercury* (Macrae *et al.*, 2006 ▶); software used to prepare material for publication: *SHELXL97*.

## Supplementary Material

Crystal structure: contains datablocks global, I. DOI: 10.1107/S1600536809045152/su2154sup1.cif

Structure factors: contains datablocks I. DOI: 10.1107/S1600536809045152/su2154Isup2.hkl

Additional supplementary materials:  crystallographic information; 3D view; checkCIF report

## Figures and Tables

**Table 1:** Hydrogen-bond geometry (Å, °)

*D*—H⋯*A*	*D*—H	H⋯*A*	*D*⋯*A*	*D*—H⋯*A*
O11—H11*O*⋯N12^i^	0.94 (3)	1.83 (3)	2.749 (3)	167 (3)
N12—H12*B*⋯O11^ii^	0.96 (3)	2.09 (3)	3.046 (3)	171 (2)

Symmetry codes: (i) 
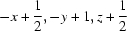
; (ii) 
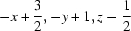
.

**Fig. 1.**
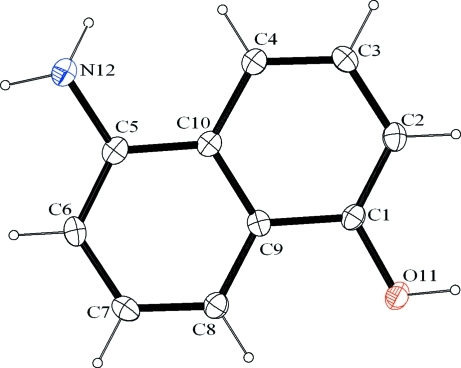
The molecular structure of the title compound, with displacement ellipsoids shown at the 30% probability level.

**Fig. 2.**
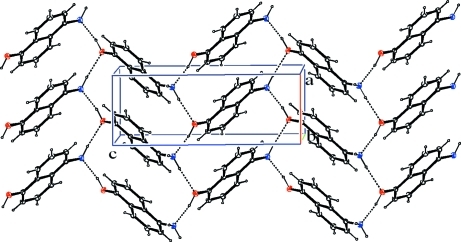
The crystal packing, viewed along the b-axis, of the title compound, showing the two-dimensional hydrogen-bonded assembly (hydrogen bonds are shown as dashed lines).

## supplementary crystallographic information

### Comment

Amino-hydroxy group recognition has been at the focus of crystal engineering since Ermer & Eling (1994) and Hanessian *et al.* (1994) noticed the complementarity of hydroxy and amino groups as regards hydrogen-bond donors and acceptors. Shortly after it was demonstrated with simple 1,2- and 1,3-aminophenols that molecular features, such as functional groups, do not necessarily result in a single manner of the molecular arrangement, and that strong N—H···O and O—H···N hydrogen bonding is frequently unable to exclude other factors from controlling the crystal packing (Allen *et al.*, 1997; Dey *et al.* 2005). Here we report on the crystal structure of a simple aminonaphthol which, as regards the substitution pattern, can be considered as an analogue of 1,4-aminophenol.

The molecular structure of the title compound is shown in Fig. 1, and the geometrical parameters are available in the archived CIF. Whereas 1,4-aminophenol forms a tetrahedral network, *via* N—H···O and O—H···N hydrogen bonds (Ermer & Eling, 1994), in the title compound the molecules are connected *via* chains of ···N—H···O—H··· interactions to form a two-dimensional polymeric structure (Fig. 2, Table 1). The two-dimensional assembly of the molecules joined by hydrogen bonding resembles that formed by 1,5-dihydroxynaphthalene (Belskii *et al.*, 1990). One amino H-atom is not involved in hydrogen bonding and does not take part in any other specific interactions.

### Experimental

Single crystals of the commercially available 5-amino-1-naphthol (Aldrich) were obtained from chloroform solution by slow evaporation.

### Refinement

In the absence of significant anomalous scattering effects, Friedel pairs were averaged. All H-atoms were located in electron-density difference maps. The H-atoms of the OH and NH groups were freely refined: O-H = 0.94 (3) Å, N-H = 0.95 (3)- 0.96 (3) Å. The C-bound H-atoms were placed at calculated positions, with C—H = 0.93 Å, and refined as riding on their carrier C-atom, with U_iso_(H) = 1.2*U*_eq_(C). .

### Crystal data

**Table:**

C_10_H_9_NO	*D*_x_ = 1.353 Mg m^−^^3^
*M**_r_* = 159.18	Melting point: 461 K
Orthorhombic, *P*2_1_2_1_2_1_	Mo *K*α radiation, λ = 0.71073 Å
Hall symbol: P 2ac 2ab	Cell parameters from 2383 reflections
*a* = 4.8607 (2) Å	θ = 4–27°
*b* = 12.3175 (6) Å	µ = 0.09 mm^−^^1^
*c* = 13.0565 (5) Å	*T* = 130 K
*V* = 781.71 (6) Å^3^	Plate, pale pink
*Z* = 4	0.30 × 0.15 × 0.02 mm
*F*(000) = 336	

### Data collection

**Table:**

Kuma KM-4-CCD κ-geometry diffractometer	726 reflections with *I* > 2σ(*I*)
Radiation source: fine-focus sealed tube	*R*_int_ = 0.037
graphite	θ_max_ = 26.4°, θ_min_ = 4.5°
ω scans	*h* = −6→5
8484 measured reflections	*k* = −15→15
963 independent reflections	*l* = −16→16

### Refinement

**Table:**

Refinement on *F*^2^	Primary atom site location: structure-invariant direct methods
Least-squares matrix: full	Secondary atom site location: difference Fourier map
*R*[*F*^2^ > 2σ(*F*^2^)] = 0.036	Hydrogen site location: inferred from neighbouring sites
*wR*(*F*^2^) = 0.091	H atoms treated by a mixture of independent and constrained refinement
*S* = 1.06	*w* = 1/[σ^2^(*F*_o_^2^) + (0.0554*P*)^2^] where *P* = (*F*_o_^2^ + 2*F*_c_^2^)/3
963 reflections	(Δ/σ)_max_ = 0.001
121 parameters	Δρ_max_ = 0.17 e Å^−^^3^
0 restraints	Δρ_min_ = −0.21 e Å^−^^3^

### Special details

**Table:**

Geometry. All e.s.d.'s (except the e.s.d. in the dihedral angle between two l.s. planes) are estimated using the full covariance matrix. The cell e.s.d.'s are taken into account individually in the estimation of e.s.d.'s in distances, angles and torsion angles; correlations between e.s.d.'s in cell parameters are only used when they are defined by crystal symmetry. An approximate (isotropic) treatment of cell e.s.d.'s is used for estimating e.s.d.'s involving l.s. planes.
Refinement. Refinement of *F*^2^ against ALL reflections. The weighted *R*-factor *wR* and goodness of fit *S* are based on *F*^2^, conventional *R*-factors *R* are based on *F*, with *F* set to zero for negative *F*^2^. The threshold expression of *F*^2^ > σ(*F*^2^) is used only for calculating *R*-factors(gt) *etc*. and is not relevant to the choice of reflections for refinement. *R*-factors based on *F*^2^ are statistically about twice as large as those based on *F*, and *R*- factors based on ALL data will be even larger.

### Fractional atomic coordinates and isotropic or equivalent isotropic displacement parameters (Å^2^)

**Table:**

	*x*	*y*	*z*	*U*_iso_*/*U*_eq_	
C1	0.2605 (5)	0.48405 (18)	0.50574 (15)	0.0232 (5)	
C2	0.1125 (5)	0.57846 (19)	0.51109 (17)	0.0269 (6)	
H2	−0.0135	0.5887	0.5638	0.032*	
C3	0.1504 (5)	0.65964 (19)	0.43738 (17)	0.0268 (6)	
H3	0.0466	0.7230	0.4409	0.032*	
C4	0.3386 (5)	0.64703 (19)	0.35998 (17)	0.0248 (6)	
H4	0.3630	0.7022	0.3122	0.030*	
C5	0.6956 (5)	0.53275 (18)	0.27339 (16)	0.0239 (6)	
C6	0.8417 (5)	0.43775 (19)	0.26935 (17)	0.0265 (6)	
H6	0.9701	0.4269	0.2175	0.032*	
C7	0.7983 (5)	0.35673 (19)	0.34304 (17)	0.0283 (6)	
H7	0.9009	0.2931	0.3399	0.034*	
C8	0.6085 (5)	0.36936 (19)	0.41937 (17)	0.0256 (6)	
H8	0.5795	0.3141	0.4667	0.031*	
C9	0.4571 (5)	0.46670 (19)	0.42580 (16)	0.0215 (5)	
C10	0.4964 (5)	0.55036 (18)	0.35220 (16)	0.0215 (5)	
O11	0.2370 (4)	0.40173 (13)	0.57550 (11)	0.0289 (4)	
H11O	0.067 (7)	0.404 (2)	0.609 (2)	0.058 (10)*	
N12	0.7265 (5)	0.61211 (18)	0.19544 (14)	0.0275 (5)	
H12A	0.699 (6)	0.685 (2)	0.2155 (18)	0.044 (8)*	
H12B	0.899 (6)	0.601 (2)	0.1610 (19)	0.038 (8)*	

### Atomic displacement parameters (Å^2^)

**Table:**

	*U*^11^	*U*^22^	*U*^33^	*U*^12^	*U*^13^	*U*^23^
C1	0.0231 (12)	0.0279 (13)	0.0186 (10)	−0.0035 (12)	−0.0020 (11)	0.0014 (10)
C2	0.0250 (13)	0.0332 (14)	0.0226 (11)	−0.0002 (12)	0.0011 (11)	−0.0024 (11)
C3	0.0261 (13)	0.0255 (13)	0.0288 (12)	0.0028 (12)	−0.0008 (11)	−0.0047 (11)
C4	0.0250 (13)	0.0272 (13)	0.0223 (11)	−0.0030 (11)	−0.0017 (11)	0.0001 (10)
C5	0.0244 (14)	0.0283 (13)	0.0192 (10)	−0.0070 (11)	−0.0053 (10)	−0.0014 (10)
C6	0.0232 (13)	0.0334 (13)	0.0229 (11)	0.0006 (12)	0.0024 (10)	−0.0056 (11)
C7	0.0294 (15)	0.0244 (13)	0.0311 (12)	0.0055 (12)	−0.0022 (11)	−0.0044 (11)
C8	0.0273 (13)	0.0278 (13)	0.0216 (11)	−0.0011 (11)	−0.0013 (11)	−0.0004 (10)
C9	0.0187 (12)	0.0263 (13)	0.0194 (10)	−0.0006 (11)	−0.0030 (10)	−0.0036 (10)
C10	0.0204 (12)	0.0249 (13)	0.0191 (10)	−0.0044 (11)	−0.0044 (10)	−0.0006 (10)
O11	0.0269 (9)	0.0356 (10)	0.0243 (8)	−0.0003 (9)	0.0036 (8)	0.0061 (8)
N12	0.0271 (12)	0.0316 (13)	0.0237 (10)	−0.0025 (12)	0.0005 (10)	0.0016 (9)

### Geometric parameters (Å, °)

**Table:**

C1—O11	1.368 (2)	C5—C10	1.430 (3)
C1—C2	1.369 (3)	C6—C7	1.402 (3)
C1—C9	1.431 (3)	C6—H6	0.9300
C2—C3	1.400 (3)	C7—C8	1.367 (3)
C2—H2	0.9300	C7—H7	0.9300
C3—C4	1.372 (3)	C8—C9	1.409 (3)
C3—H3	0.9300	C8—H8	0.9300
C4—C10	1.420 (3)	C9—C10	1.422 (3)
C4—H4	0.9300	O11—H11O	0.94 (3)
C5—C6	1.370 (3)	N12—H12A	0.95 (3)
C5—N12	1.419 (3)	N12—H12B	0.96 (3)
			
O11—C1—C2	123.5 (2)	C7—C6—H6	119.9
O11—C1—C9	115.5 (2)	C8—C7—C6	121.4 (2)
C2—C1—C9	120.99 (19)	C8—C7—H7	119.3
C1—C2—C3	120.2 (2)	C6—C7—H7	119.3
C1—C2—H2	119.9	C7—C8—C9	119.5 (2)
C3—C2—H2	119.9	C7—C8—H8	120.2
C4—C3—C2	120.9 (2)	C9—C8—H8	120.2
C4—C3—H3	119.6	C8—C9—C10	120.4 (2)
C2—C3—H3	119.6	C8—C9—C1	121.3 (2)
C3—C4—C10	120.5 (2)	C10—C9—C1	118.3 (2)
C3—C4—H4	119.7	C4—C10—C9	119.1 (2)
C10—C4—H4	119.7	C4—C10—C5	123.0 (2)
C6—C5—N12	120.4 (2)	C9—C10—C5	117.9 (2)
C6—C5—C10	120.6 (2)	C1—O11—H11O	111.6 (18)
N12—C5—C10	118.9 (2)	C5—N12—H12A	116.2 (15)
C5—C6—C7	120.2 (2)	C5—N12—H12B	109.1 (15)
C5—C6—H6	119.9	H12A—N12—H12B	113 (2)

### Hydrogen-bond geometry (Å, °)

**Table:**

*D*—H···*A*	*D*—H	H···*A*	*D*···*A*	*D*—H···*A*
O11—H11O···N12^i^	0.94 (3)	1.83 (3)	2.749 (3)	167 (3)
N12—H12B···O11^ii^	0.96 (3)	2.09 (3)	3.046 (3)	171 (2)

Symmetry codes: (i) −*x*+1/2, −*y*+1, *z*+1/2; (ii) −*x*+3/2, −*y*+1, *z*−1/2.
